# Supplementary material for: Comparison of Toxicity and Cellular Uptake of CdSe/ZnS and Carbon Quantum Dots for Molecular Tracking Using Saccharomyces cerevisiae as a Fungal Model
Source: Nanomaterials (Basel). 2023 Dec 19;14(1):10. doi: 10.3390/nano14010010 (PMC10781119; doi:10.3390/nano14010010)
Supplement: Supplementary file 1 [file nanomaterials-14-00010-s001.zip › nanomaterials-2735600-supplementary.pdf]

Supporting Information

Comparison of toxicity and cellular uptake of CdSe/ZnS and carbon quantum dots for molecular tracking using *Saccharomyces cerevisiae* as a fungal model

Sanni M. A. Färkkilä[1], Monika Mortimer[2], Raivo Jaaniso[3], Anne Kahru[2], Valter Kiisk[3], Arvo Kikas[3], Jekaterina Kozlova[3], Imbi Kurvet[2], Uno Mäeorg[4], Maarja Otsus[2], Kaja Kasemets[2]

[1] Institute of Ecology and Earth Sciences, University of Tartu, Juhan Liivi 2, 50409 Tartu, Estonia

[2]Laboratory of Environmental Toxicology, National Institute of Chemical Physics and Biophysics, Akadeemia tee 23, Tallinn 12618, Estonia

[3] Institute of Physics, University of Tartu, W. Ostwaldi 1, 50411 Tartu, Estonia

[4] Institute of Chemistry, University of Tartu, Ravila 14a, 50411, Tartu, Estonia

Table S1. A summary table about the findings of articles dealing with Cd-containing quantum dots or carbon quantum dots and yeast. NA denotes that the information is not provided, and ND denotes not determined in the study. Literature was reviewed using a combination of systematic quarry (Document search from Web of Science, keywords "quantum dot" OR "carbon dot" AND yeast* OR Saccharomyces OR Candida) and citation searching. Relevant primary research papers from journals with impact factor >1 were included in the table.

Particle type	Size	Modification	Yeast	Concentration	Exposure time	Toxicity	Interaction	Publication
CdS	1-7 nm	None	*Saccharomyces cerevisiae*	10–30 mg/L	Up to 15h	Concentrations > 10 mg/L affected growth and extracellular proteins	Entry assumed but not visually shown	[78]
CdS	5 nm	None	*S. cerevisiae*	40-1000 mg/L	Up to 72h	Growth was negatively affected at 250-500 mg/L depending on growth media; numerous biological	QDs were added with nystatin, which produces pores in the cell membranes to facilitate entry, but it was not	[71]

						processes were affected via gene expression. Toxicity was not explained by Cd release	confirmed whether they entered cells	
CdS	<5 nm	None	*S. cerevisiae*	100 mg/L (selected to be sublethal)	NA	The sublethal concentration negatively affected mitochondrial processes and enzyme activity	ND	[72]
CdS	5 nm	None	*S. cerevisiae*	Up to 100 mg/L	Up to 24h	QDs were toxic and changed the gene expression of many essential genes; toxicity of QDs was not caused solely by the Cd	QDs were added together with nystatin, which produces pores in the cell membranes to facilitate entry, but it was not confirmed whether they entered the cells.	[73]
CdS	0.36 nm, aggregated to 50-100 nm	None	*S. cerevisiae*	75-150 mg/L (selected to be sublethal)	Up to 24h	Even the lowest tested concentration caused toxic responses related to cell mitochondria and reactive oxygen species (ROS) generation	QDs were added together with nystatin, which produces pores in the cell membranes to facilitate entry, but it was not confirmed whether they entered the cells.	[82]
CdS	5 nm	None	*S. cerevisiae*	Up to 100 mg/L	Up to 24h	Concentrations > 10 mg/L strongly reduced cell viability, even 3 mg/L affected the viability of some strains at 24h exposure, sporulation was also negatively affected	ND	[80]

CdS	5 nm	None	*S. cerevisiae*	0.1-250 mg/L	48h	Higher concentrations negatively affected the growth of the wild-type strain, strains with mutations related to corona protein formation were more tolerant 2.5 mg/L QDs negatively affected enzyme activity	ND	[81]
CdSe	3-10 nm	MAA[i] coated, WGA[ii] or transferrin conjugated	*Schizosaccharomyces pombe*	250 nM	8h	ND	No interaction	[82]
CdSe	20-30 nm	None	*S. cerevisiae*	Up to 160 mg/L	12h	IC50[iii] at 80 mg/L	Entry assumed but not visually shown	[83]
CdSe	4.0 and 3.8 nm	MHDA[iv]-coated	*S. cerevisiae*	500nM for adaptation experiment	Up to 24 days	Initial MIC[v] were 1300 nM and 950 nM depending on the particle, but cells adapted to long exposure, raising MIC values up to 10-fold. Adapted cells did not tolerate Cd ions much better than control cells	QDs reported to enter cells, data not shown	[60]
CdSe/ZnS	39.47 nm (hydrodynamic)	polymer coated	*S. cerevisiae*	5 nM	24h	ND for yeast	Adsorption of particles to cell surface, the amount of adsorbed particles reduced to ~7% and ~0.7% after	[59]

							one and two washes	
CdSe/ZnS	NA	Carboxyl capped, cysteamine conjugated	*S. cerevisiae*	NA	Up to 360 min	No toxicity observed	Cells were treated with TCEP[vi] to create free SH-groups on the cell walls for QD binding. QDs bound to the cell walls and were transferred to the cell surfaces of daughter cells	[84]
CdSe/ZnS	6.1–9.5 nm	None	*S. cerevisiae*	10-100 mg/L	Up to 24h	No adverse effect on growth (growth stimulation at 20-100 mg/L), but changes in gene expression and ROS generation	ND	[85]
CdSe/ZnS	4.1 nm (estimation)	None	*S. cerevisiae*	5-100 mg/L	Up to 24 h	No adverse effect on growth or ROS generation, but changes in gene expression	ND	[86]
CdSe/ZnS	5-10 nm	Carboxyl capped	*S. cerevisiae*	4-50 mg/L	Up to 24h	All concentrations lengthened the lag phase. Higher concentrations changed gene expression and cellular processes	QDs attached to cell surfaces, mostly to mother cells, and were later taken in primarily by endocytosis	[87]
CdSe/ZnS	3.86 ± 0.17 nm and 3.96 ± 0.14 nm	Polymer coated	*S. cerevisiae*	Up to 2000 nM	Up to 18h	Minor influence on cell wall morphology	Adhesion to cell surface, more adhesion when cell density was high, and in budding zones	[88]
CdSeS/ZnS	~5.6 nm	GSH[vii] conjugated	*S. cerevisiae*	11 050 000 nM	23h	ND	Uptake by cells and transfer to daughter cells via cytoplasm	[61]

CdSe/ZnS	308 ± 150 nm (hydrodynamic) for CdSe/ZnS-Cys	Carboxyl capping and cysteine conjugation	*S. cerevisiae*	Up to 100 mg/L	Up to 24h	Minimum biocidal concentration (MBC) of all particles was >100 mg/L	CdSe/ZnS QDS with or without cysteine conjugation adsorbed to cell surfaces without cell entry	The current work
CdTe	~2.5 nm	Thioglycolic acid (TGA capped), Mannose or Galactose or Glucose conjugated	*Kluyveromyces bulgaricus, S. cerevisiae*	NA	15 mins	ND	Unconjugated QDs were weakly bound to cells, conjugated cells more strongly bound to cells depending on yeast sugar preference. Entry not reported	[89]
CdTe	~3.6 nm	Lectin conjugated, MSA[viii] capped	*Candida albicans*	NA	1h	ND	Lectin-conjugated QDs adsorb to the surfaces of cells, adsorption efficiency was affected by pH (the best efficiency, 92% of cells labelled, was at pH 7.0). Unconjugated QDs only rarely adsorbed after washing	[90]
CdTe	4.1 ± 0.152 nm	NAC[ix], MAA or GSH conjugated	*S. cerevisiae*	Up to 312 nM	6h	Particles are toxic with IC50 from ~15nM to ~90nM, in order GSH-CdTe QDs < MAA-CdTe QDs < NAC-CdTe QDs. All types alter cell	All particles reportedly enter yeast cells	[62]

						morphology and various other properties		
CdTe	2.8 nm	Lectin conjugated, MSA capped	*C. albican*s, *C. glabrata*, *C. parapilopsis*	NA	1h	ND	QDs conjugated to lectin labeled the cells by covering the cell surfaces, allowing the observations of differences in cell size and shape as well as carbohydrate composition of cell walls between Candida species	[91]
CdTe	3-4 nm	Lectin conjugated, MSA capped	*C. albicans*	5000 nM	1h	ND	QDs conjugated to lectin labeled the cells by covering the cell surfaces	[92]
CdTe	~2.3 nm	None	*S. cerevisiae*	6-60 nM	Up to 14h	QDs disrupted cell membranes and growth was negatively affected already at 6nM	ND	[93]
CdTe	4.1±0.152 nm and 5.8±0.152 nm	NAC-capped	*S. cerevisiae*	Up to 867.2 nM	Up to 16h	IC50 59.44 nM and 186.61 nM depending on size, smaller particles are reportedly less toxic	ND	[94]
CdTe	4.1±0.152 nm and 5.8±0.152 nm	NAC-capped	*S. cerevisiae*	Up to 209.4 nM	6-12 h	IC50 17.07 nM and 80.81 nM depending on size, smaller particles are reportedly less toxic	Particles enter cells and induce breakage of the cells	[63]
CdTe-CdS	3–7 nm	MAA-capped and	Yeast (species not reported)	NA	Up to 7 min	ND	Glucose-conjugated QDs enter cells, while	[64]

		glucose conjugated					unconjugated ones do not	
CQDs (citric acid and cysteine)	6.1 ± 3.35 nm	None	*S. cerevisiae*	Up to 100 mg/L	Up to 24h	Minimum biocidal concentration (MBC) of all particles was >100 mg/L	Particles entered cells and distributed into the cytoplasm	The current work
CQDs (biological matter)	5 ± 2 nm	None	*C. albicans*	NA	Up to 24h		CQD solution stained the yeast cytosol	[95]
CQDs (biological matter)	~2.5 nm	None	*S. cerevisiae*	NA	Up to 24h		Particles entered cells and distributed homogeneously	[96]
CQDs (bottom-up from citric acid and PEG[x])	12 ± 1 (hydrodynamic)	None	*C. albicans*	25-200 mg/L	Up to 48h	MIC80[xi] at 62.5 mg/L for the smallest particle size, larger particles extended the lag-phase	Particles were presumably (uptake not confirmed) taken up by cells and caused growth inhibition, potentially via ROS formation	[97]
CQDs (citric acid pyrolysis)	3.1 ± 0.4 nm	None	*Pichia pastoris*	Up to 25 000 mg/L	3-16h	Dose-dependent reduction of cell growth in concentrations >10 000 mg/L	Adsorption of particles to the cell surface, more adsorption with a larger dose	[98]
CQDs (Citric acid, ethylene diamine and boric acid)	7.5–8.5 nm	None	*S. cerevisiae*	50-300 mg/L	NA	ND	Dead yeast cells were intensely fluorescent throughout, while live cells were only fluorescent on the surface. Authors assume NPs are too big to enter live cells but not dead ones	[99]

CQDs (citric acid, ethylene diamine, and boric acid)	~4 nm	None	Yeast (species not reported)	Up to 400 000 mg/L	Up to 10h	No negative effects on growth at concentrations up to 400 000 mg/L	1 minute incubation coated the outsides of all cells and insides of dead cells. Thus, it was possible to differentiate dead and live yeast cells by CQD fluorescence. Increasing concentration and time increased the fluorescence intensity of cells	[100]
CQDs (top-down from biological matter)	~3 nm	None	*S. cerevisiae*	40 mg/L (for uptake)	1-6h	No observed toxicity in concentrations up to 400 mg/mL	Particles were taken up by cells and homogeneously distributed within them	[101]
CQDs (citric acid + 4 different precursors)	6.8 nm, 17.9 nm and 36.4nm	None	*S. cerevisiae*	50, 25, 10, 2, and 1 % of the final volume	Up to 48h	Toxicity was slightly dependent on the precursors, but generally high CQD concentrations reduced growth, also pH affected toxicity	Only particular varieties of CQDs bound to the cell surface and were taken up	[102]
GQDs (graphene oxide)	NA	None	*S. cerevisiae*	NA for yeast	NA for yeast	ND for yeast	Entry to cells reported	[103]

[i] MAA – mercaptoacetic acid
[ii] WGA – wheat germ agglutinin
[iii] IC50, 50% inhibitory concentration – the concentration in which the growth of 50% of the cells is inhibited
[iv] MHDA – mercaptohexadecanoic acid
[v] MIC – minimum inhibitory concentration – the smallest concentration that fully inhibits the growth of cells

[vi] TCEP – tris(2-carboxyethyl)phosphine
[vii] GSH – glutathione
[viii] MSA – 3-mercaptosuccinic acid
[ix] NAC – N-acetyl cysteine
[x] PEG – Polyethylene glycol
[xi] MIC80, 80% minimum inhibitory concentration – the smallest concentration that inhibits the growth of 80% of the cells.

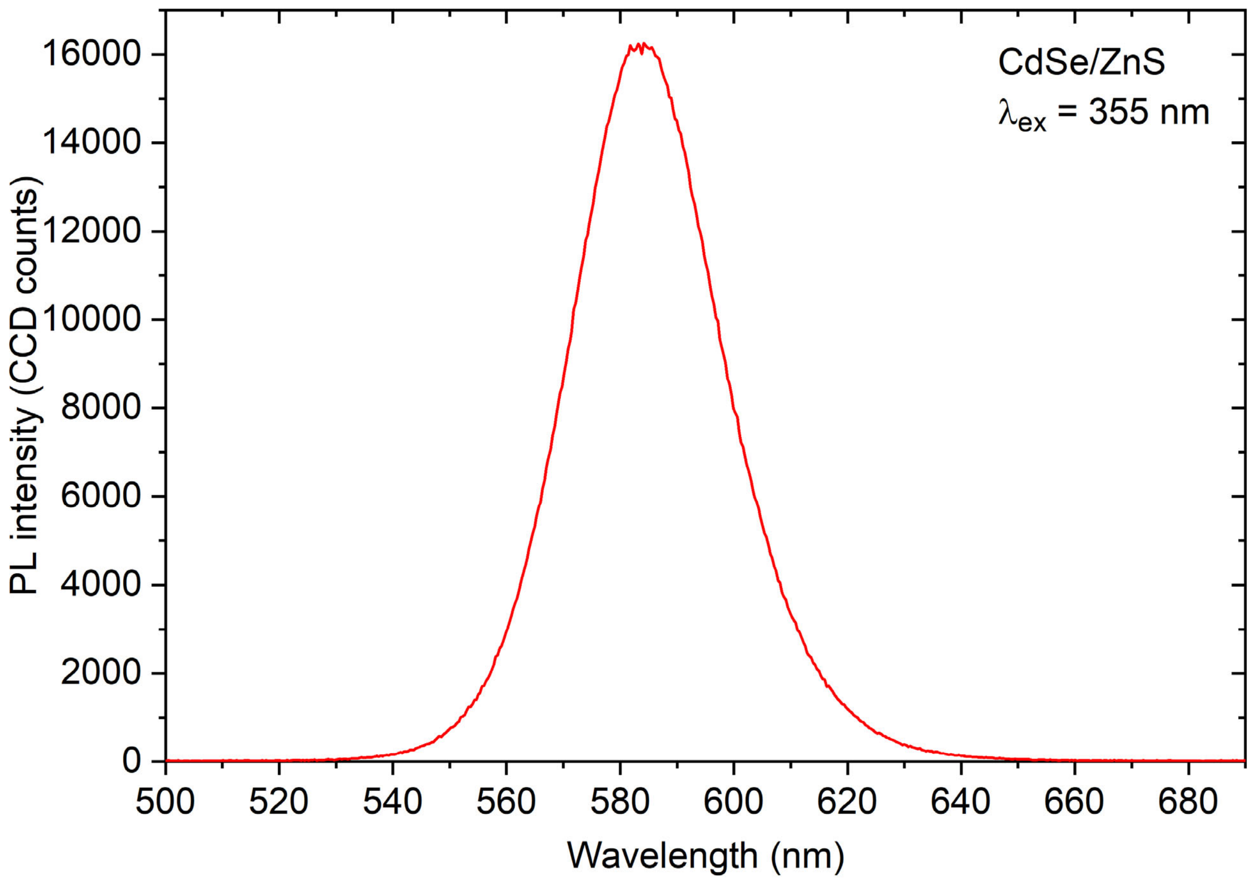

Figure S1. Fluorescence spectrum of CdSe/ZnS QDs.

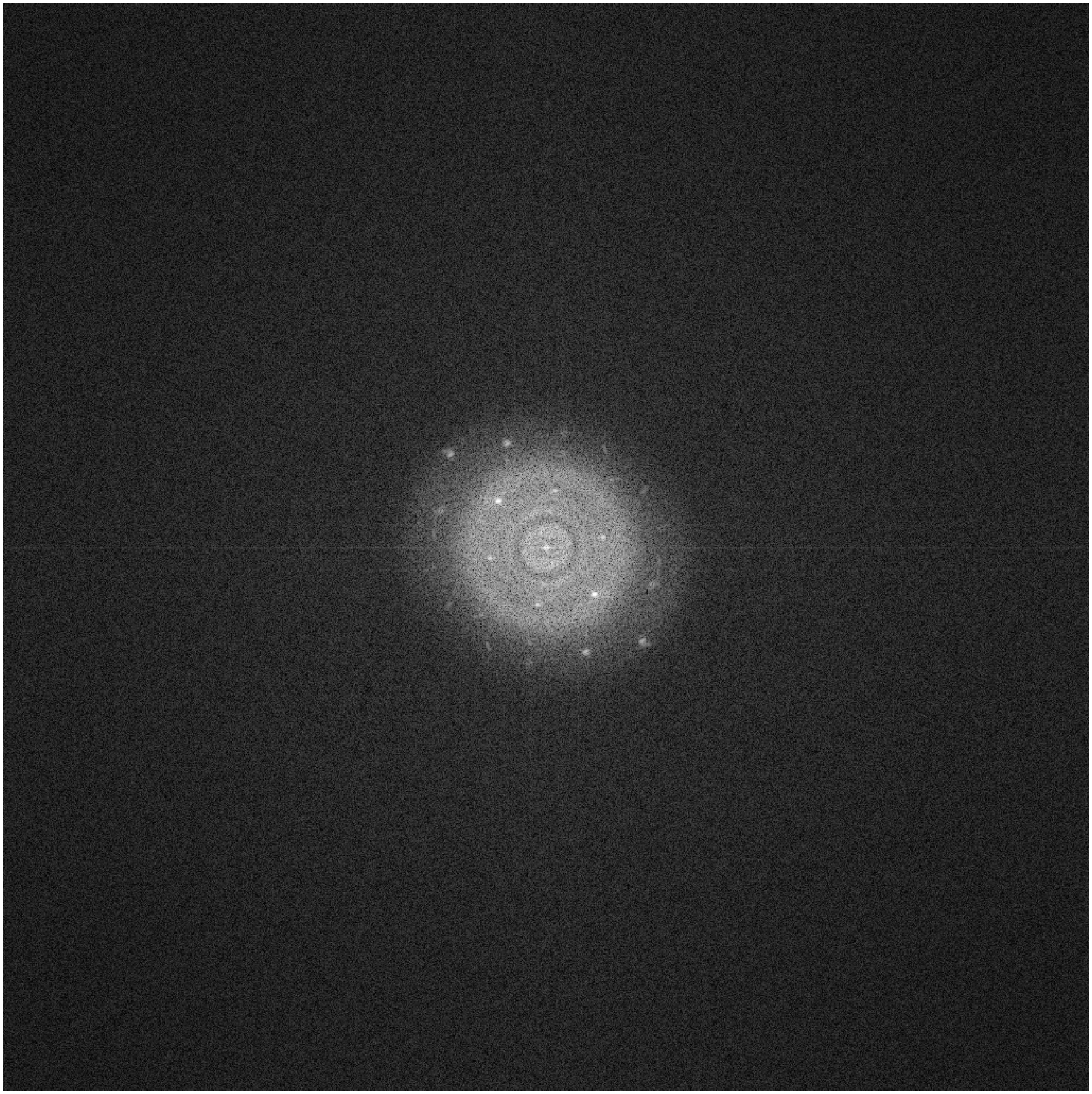

Figure S2. The Fourier transform image of the crystalline region characterizing the reciprocal lattice of CdSe/ZnS QDs.
